# Stakeholder perspectives on integration of mental health services into primary care: a mixed methods study in Northern Iraq

**DOI:** 10.1186/s13033-019-0330-7

**Published:** 2019-12-28

**Authors:** Amanda J. Nguyen, Natalie Rykiel, Laura Murray, Ahmed Amin, Emily Haroz, Catherine Lee, Paul Bolton

**Affiliations:** 1 Youth-Nex and the Department of Human Services, Curry School of Education and Human Development, University of Virginia, Charlottesville, VA 22904 USA; 2 Division of Pulmonary and Critical Care, Johns Hopkins University School of Medicine, Baltimore, MD 21205 USA; 3 Department of Mental Health, Johns Hopkins Bloomberg School of Public Health, Baltimore, MD 21205 USA; 4 Wchan Organization for Victims of Human Rights Violations & Sulaimani Polytechnic University–Technical College of Health, Sulaymaniyah, Iraq; 5 Department of International Health, Johns Hopkins Bloomberg School of Public Health, Baltimore, MD 21205 USA

**Keywords:** Mental health, Implementation, Iraq, Primary care

## Abstract

**Background:**

Integrating evidence-based mental health services into primary care has been identified as one strategy for overcoming the treatment gap in low and middle-income countries, yet their uptake into standard practice remains poor. The purpose of this study was to understand stakeholder perspectives regarding barriers and facilitators to integration of mental health services into primary care settings in Northern Iraq.

**Methods:**

Using a convergent mixed methods study design, quantitative and qualitative questionnaires assessed respondent perceptions of implementation factors under the domains of Autonomy, Acceptability, Appropriateness, Feasibility, Penetration/Accessibility, Sustainability, and Organizational Climate. We interviewed four types of stakeholders: clients, providers of mental health services, non-mental health (MH) staff working at the centers, and center directors. Interviews were conducted with clients at the completion of services, and with all other stakeholder groups in the latter half of the first year of program implementation, by Kurdish-speaking interviewer pairs. Qualitative and quantitative data were analyzed separately and merged using qualitative data transformation to quantify frequency of theme and integrate with quantitative findings through woven narrative.

**Results:**

123 clients, 26 providers, 40 non-MH staff, and 12 directors provided data. Positive perceptions of the program’s acceptability, appropriateness, feasibility, and positive impacts were reported across all stakeholder levels. Providers reported that the program length (8–12 sessions) was a challenge. Clients described logistical challenges (e.g.: transportation, childcare, home duties); support from family and friends appeared to be critical. Lack of private space, insufficient staffing, and need for greater government support were also important issues.

**Conclusions:**

This mixed methods study is unique in its inclusion of non-MH staff and director perspectives on integration of mental health services in primary care clinics. Their inclusion proved vital since they included critical human resource barriers to feasibility. Providers reported generally positive integration experiences but that some colleagues (clinic staff not involved in mental health services) were unsupportive. Most non-MH staff were supportive, but some did report negative impacts on their working environment. Future studies of integration of mental health services into other service platforms should include the perspectives of stakeholders not involved in provision of mental health services.

## Introduction

Iraq has experienced decades of human rights abuses, armed conflict, economic crisis and political instability resulting in serious mental health problems among trauma survivors [1]. It is estimated that nearly 20% of the Iraqi population will experience a mental health problem over the life course, most commonly anxiety disorders, post-traumatic stress disorder (PTSD), and depression [2]. Epidemiological study also indicates increasing prevalence of common mental disorders (CMDs), with a particularly notable rise in panic disorders and PTSD [2]. Historically, mental health services in Iraq have largely been provided by psychiatrists in medical facilities [3], resulting in a large gap between mental health need and service availability [4]. In the late 2000s, the Iraq Ministry of Health began initiatives to rebuild and decentralize mental health services, including integration of mental health services into primary care [3, 5].

Despite increasing evidence for the effectiveness of psychotherapeutic treatments for common mental disorders (CMDs) such as depression, anxiety, and PTSD in low-and middle-income countries (LMIC) [6] uptake of these interventions into standard practice remains poor [7]. Integrating mental health services into primary care has been identified as one possible strategy for overcoming the treatment gap in LMIC, with potential benefits including increased access, more holistic care, stigma reduction, and system strengthening [8]. In order to achieve integration, it is important to first understand the facilitators and barriers faced by stakeholders, and how these vary in different contexts.

In 2008, the Applied Mental Health Research Group (AMHR) at Johns Hopkins University, and Heartland Alliance International began development of integrating evidence-based mental health services in Iraq. This included several RCTs conducted between 2009 and 2012 to evaluate the effectiveness of different treatments (e.g., CPT, Behavioral Activation) in Iraq for symptoms of depression, anxiety, and post-traumatic stress [9–11]. From these studies, the Common Elements Treatment Approach (CETA) was found to be most effective, as well as acceptable to providers and clients [9–11]. CETA is a modular, multi-problem transdiagnostic treatment approach for CMDs which consists of 8–12 weekly hour-long sessions based on cognitive behavioral psychotherapy and delivered by lay providers [12].

On the strength of these trial results for CETA, Heartland Alliance and Wchan (a newly formed local NGO) planned to scale up CETA services in primary health centers operated by the Ministry of Health beginning in 2013. As in the trial, existing clinical staff (e.g.: nurses, pharmacists) from these clinics were trained as CETA providers and, as part of their weekly work load, some time each week would be allocated to providing CETA while they continued to serve in their previous roles. Following the apprenticeship model, local supervisors that had additional training met weekly with providers to review cases and consult on following CETA [13].

Throughout the CETA scale up efforts of Heartland Alliance and Wchan, our team conducted a parallel implementation study to understand stakeholder perspectives regarding barriers and facilitators to mental health service uptake and sustainability when integrated into primary care settings. Unlike most other implementation research, we focused not only on providers and clients but also on other health center staff and directors in the clinics in which CETA was introduced. By taking into account the views and needs of all four stakeholder levels, we aimed to inform the building of mental health services that are sustainable and acceptable to all stakeholders.

## Methods

### Setting

This study took place from January through December 2014, during the first year roll-out of CETA services after completion of the previously described RCTs. While CETA scale-up efforts extended across other areas of Iraq as well, sites chosen for this implementation research were clinics in areas of Northern Iraq where Wchan provided services and was able to lead the research. Twenty-six CETA providers (22 counselors and four supervisors) provided services in twelve clinics in and around Erbil, Sulimaniyah, and Garmyan. During planning and preparation, the political situation in Northern Iraq was stable. However, the implementation period included a major Islamic State military offensive in June of 2014, which contributed to heightened political and economic instability; this is likely to have exacerbated existing challenges for mental health service delivery, although it did not directly impact data collection activities.

### Study participants

Participants included male and female adults living in Northern Iraq who were either: (1) persons who met screening criteria for CETA (i.e. “clients”); (2) counselors and supervisors trained in CETA and responsible for service provision (i.e. “providers”); (3) staff, such office and administrative staff, working at the clinic sites in non-mental health roles (i.e. “non-MH staff”), or (4) clinic directors (i.e. “directors”).

Potential clients were referred by clinic staff to the CETA providers to be evaluated using a locally validated assessment instrument developed for use in the prior RCT [10]. Individuals who met a cut-off score indicating elevated symptoms of depression or post-traumatic stress were designated as clients, offered services, and informed about the implementation study by the provider. Those clients who agreed to be contacted about the study were then added to a contact list. Providers, non-MH staff, and directors were recruited using staffing lists. Interviewers contacted each potential participant by phone to introduce the study and arrange an in-person interview, which could be completed in one or multiple sessions based on respondent preference. Informed consent was obtained for all participants at the first in-person meeting prior to any data collection; employees and clients were assured that participation would not impact their employment or service eligibility, respectively, nor would their colleagues/supervisors or CETA provider have access to the information they provided.

All clients presenting to the clinics who were assessed and found to be eligible for CETA services were invited to participate in this study regardless of whether they decided to initiate treatment. The intent for this broader inclusion was to understand potential barriers to the full range of service uptake, including initiation as well as continued participation. All providers and clinic directors were also invited to participate, as were a convenience sample of 2–4 additional non-MH staff per clinic (some clinics were quite small and had less than four non-MH staff). The full provider, non-MH staff, and director samples contributed both qualitative and quantitative data. At the client level, only a subset of participants contributed qualitative data, in order of completion for all those agreeing to complete the interview, until saturation was reached [14] as determined by team review and discussion of transcripts.

### Instruments

Based on previous experience indicating that existing implementation instruments did not fit well with the Iraq cultural and health service delivery context, we developed a new set of semi-structured (qualitative) interview guides and quantitative instruments for this study based on three leading implementation frameworks, chosen because together they addressed both stages of implementation and multi-level contexts [15–17]. A comprehensive description of the instrument development process and resulting psychometrics is available elsewhere [18]. Briefly, the quantitative instruments were developed by operationalizing implementation domain definitions [19], consulting leading theoretical frameworks [15–17], utilizing logframes to generate indicators, drafting the instruments, and external expert review [18], with the purpose of generating informative indicators at the item level. The semi-structured interview guides consisted of open-ended questions with specific follow-up probes. Three separate sets of instruments were developed for clients, providers, and non-MH staff and directors. Despite administering the same instruments to the latter two groups, we treated these as distinct and separate stakeholder groups for analysis. All qualitative and quantitative data collection tools were translated, back-translated, and piloted prior to data collection with each stakeholder level (clients, providers, non-MH staff, and clinic directors). Adjustments were made to the translation and phrasing based on pilot feedback to improve clarity; for example, adding “in your opinion” due to respondent confusion about whether items were seeking individual or majority opinion. Sample data collection instruments are available as Additional files 1 and 2.

#### Quantitative

The quantitative instruments included demographic questions as well as indicators of theoretically relevant implementation science domains: Acceptability, Appropriateness, Feasibility, Penetration/Accessibility, and Sustainability [20]. We also included indicators of Autonomy; though less commonly considered as an implementation science domain, we theorized it may be particularly relevant when examining help-seeking in a cultural context in which gender roles may reduce autonomy [21]. For providers, non-MH staff, and directors, additional items were also included to assess Organizational Climate [22]. For most items, response options were on a 4-point Likert scale ranging from *0* “not at all” to *3* “a lot”. The instruments included 38 questions for clients, 76 for providers, and 62 for non-MH staff and directors. Although categorized into theoretical domains, the data for this paper are analyzed and reported at the item level, not as scales.

#### Qualitative

The client qualitative interview explored thoughts and opinions about CETA services in particular and the clinic more generally; what people in the community think about the services; how family and friends influenced use of the services; who makes decisions regarding their health and how these decisions are made; arrangements clients had to make to attend services and barriers and facilitators to treatment attendance; what they liked/did not like/would change about the services; how the services fit with their values and needs; how the services may have benefitted or harmed them; and anything else they felt it was important to share about the services. Although potential clients who elected not to seek treatment were not expected to have informed answers to all these topics, they remained eligible for the study as it was thought that they would provide valuable information about community perceptions, access, social influences, perceptions about fit to values and needs, and other potential barriers to care.

Provider, non-MH staff, and director qualitative interviews explored the current situation regarding mental health and mental health care in their community; challenges and facilitators to the implementation of these services, including positive aspects, gaps or challenges, and what needs to change; perceptions about the services among colleagues at the clinic where they work; perceptions about the organizational climate at their clinic (both environmental and interpersonal); specific thoughts regarding the feasibility, acceptability, sustainability, and appropriateness of the service, including barriers and facilitators for each domain as well as things that need to change to improve each domain (e.g.: What makes it less acceptable? What makes it more acceptable? What needs to change to make it more acceptable?); and anything else they felt it was important to share. Providers were also asked how they came to be recruited to provide services. Directors were asked about their ability to adjust or change the way their clinic offers services to meet community needs.

### Data collection procedures

Twenty locally based interviewers were hired by Wchan specifically for data collection (with no other role in CETA implementation). Interviewers completed a multi-day, in-person training on research ethics and interview techniques led by two members of the AMHR research team. Training included didactic instruction, discussion, modeling, role-plays, and feedback. Prior to beginning data collection, all interviewers then conducted pilot interviews with each category of stakeholder for training purposes, and participated in an additional multi-day pilot feedback meeting in which they discussed and received further training to address specific challenges encountered.

To ensure all stakeholders had the opportunity for adequate exposure to the program, data collection took place during the latter half of the implementation period, between June and December 2014. Both forms of data were collected concurrently, with the intention that the qualitative data be used to complement and expand on the quantitative data. Client interviews were conducted following completion of (or disengagement from) treatment. Interviews took place in a quiet, private location of the participant’s choice, were primarily conducted in Kurdish, and took approximately 2 h total per participant. Qualitative interviews were conducted by interviewer pairs; one led the conversation while the other transcribed the interview. No identifying information was collected; all study materials included only a study ID number.

Following each interview, the interview team worked together to type and ensure the accuracy of the transcript. A selection of transcripts—generally the first 2–3 transcripts from each interview team—were reviewed by members of the study team for quality and training feedback, which primarily centered on identifying areas where further probing would have been helpful and providing suggested probes. The supervisor reviewed this feedback with the interview teams, including additional role-play practice.

This study was approved by the Institutional Review Board at the Johns Hopkins Bloomberg School of Public Health (#5286) and a local Ethics Committee at the University of Sulimaniyah.

### Analysis

Qualitative data analysis was completed in two phases. The initial analysis was done on paper in Kurdish by the interviewers and project supervisor following the AMHR DIME [23] approach. Interview teams reviewed all transcripts and extracted key response points from each, producing a table of responses for each question at each stakeholder level. The study director and a smaller analysis team then consolidated each table by combining responses that had the same meaning and listing the number of respondents who gave each response, producing a summary Excel table of responses sorted by frequency for each question at each stakeholder level. Where different wording was used but the meaning was the same, selection of the clearest wording was made by consensus. The summary tables were then translated into English by a bilingual member of the research team, with the translation checked for accuracy by a second bilingual team member.

The second phase of analysis was done in English by the AMHR study team using the summary tables and coded responses in Excel. Each set of relevant responses was combined across all question tables, resulting in three consolidated lists (barriers, facilitators, and suggestions) per stakeholder level. Within each list, responses were then sorted and grouped according to emergent themes and sub-themes. Responses that appeared to address multiple themes were placed within both themes. Where there was uncertainty about how to code a response, the project director reviewed the original Kurdish transcript and made the decision.

Quantitative data analysis was conducted in Stata 14.1 [24]. Descriptive analysis of demographics included Chi-square and t-test comparisons. Analysis of potential barriers involved calculating individual item means to identify items with lower mean scores (i.e. less than 50% or 67% of the optimal item score) that would suggest an area of potential challenge for implementation. For most items, responses ranged from 0 to 3; we therefore considered mean scores below 1.5 to be indicative, and between 1.5 and 2 marginally indicative, of a potential barrier.

Consistent with a convergent mixed methods study design [25], qualitative and quantitative data were merged after analysis to inform interpretation following the approach of Bradt et al. [26]. Qualitative data transformation was used to quantify frequency in which each theme was discussed; these findings are then integrated with quantitative findings through woven narrative in the results [27].

## Results

### Sample description

Data is included from 123 clients (122 quantitative and 62 qualitative interviews), 26 providers (completing both interviews), 40 non-MH staff (36 quantitative, 40 qualitative), and 12 directors (completing both interviews). Demographic information is reported in Table 1; note that not all demographic variables were asked at all stakeholder levels. These samples include all providers and directors, a convenience sample of non-MH staff, and all consenting clients. No recruited non-MH staff declined to participate. The client sample comprised 48.8% of all eligible client participants (*n *= 252). Neither age, sex, marital status, nor employment status were associated with client participation (all p > .05); however, compared to the full eligible population, study participants were more likely to initiate treatment (95.9% vs. 81.0%, p < .001) and complete a clinical discharge assessment (81.2% vs. 63.2%, p = .001). Among the implementation study sample, there were no significant demographic differences between those who did and did not complete a clinical discharge assessment (all p > .05). Client respondents who had initiated treatment but did not complete a discharge assessment completed an average of 6.7 sessions (range: 2–14); only five respondents had no treatment exposure. Therefore, almost all clients interviewed were meaningfully exposed to CETA. We were not successful in obtaining the perspectives of those who had little to no service engagement.

**Table 1:** Sample description by stakeholder level

	Client *(n *= *123*^a^*)* %	Provider *(n *= *26)* %	Non-MH Staff *(n *= *40*^a^*)* %	Director *(n *= *12)* %
Age; mean (SD)	30.3 (8.4)	35.0 (7.1)	34 (6.8)	39.7 (7.8)
Gender				
Men	28.7	42.3	60	100
Women	71.31	57.7	40	0.0
Employment				
Not working	49.2	–	–	–
Irregular/daily work	12.3	–	–	–
Regular/stable work	30.3	–	–	–
Self-employed	8.2	–	–	–
Marital status				
Single	30.3	30.8	–	–
Married	65.6	69.2	–	–
Divorced	2.5	0.0	–	–
Widowed	1.6	0.0	–	–
Education				
Secondary	–	7.7	–	–
Institutional degree	–	34.6	–	–
Bachelor’s or higher	–	57.7	–	–
Location				
Erbil	13.9	19.2	35.0	41.7
Sulimaniyah	77.9	65.4	42.5	33.3
Garmyan	8.2	15.4	22.5	25.0

"–" indicates demographic variable was not assessed at that stakeholder level

^a^1 client and 4 staff did not complete the quantitative interview

### Findings

Table 2 reports quantitative survey items with mean scores less than 67% or 50% of the optimal score, indicating potential barriers identified by each stakeholder group. Qualitative data on perceived barriers, facilitators, and stakeholder suggestions for improvement are reported in Tables 3, 4, 5, respectively, and reported below as the frequency of a given response over the total number of individuals in that stakeholder group. Responses that were given by less than five individuals are not included.

**Table 2:** Quantitative Items with average scores less than 50% (●) or 67% (○) of the optimal score^a^ indicating potential barriers

Domain/item	Client	Provider	Non-MH staff	Director
***Autonomy***				
Mental health care decisions are made by others in the family	●			
***Accessibility***				
Service accessibility for most people in the community		○	○	○
Service accessibility for the poorest people in the community		○	●	●
Service accessibility for women who need them	○	○	●	○
Service accessibility for men who need them		○	○	
There are groups in the community that are unable to access the services^b^	●	●	●	●
***Acceptability***				
Services are a priority for the government		●	●	○
Services are acceptable to the community		○	○	○
Your job negatively affects your family life				○
***Feasibility***				
Difficulty attending weekly treatment sessions for 8–12 weeks	○			
Sufficient access to computer/internet equipment		●		
Sufficient access to private space to meet with clients		○	●	●
Enough counselors to implement step-by-step psychotherapy			○	○
Enough counselor *time* to implement step-by-step psychotherapy			○	○
Sufficient budget to implement step-by-step psychotherapy			●	●
Enough other necessary resources (e.g.: support staff, administrative time, transportation money, etc.)			●	●
Feasibility of integrating step-by-step psychotherapy into primary health centers			○	○
***Positive organizational structure*** *(not included in client interviews)*				
Feeling overworked		○	●	●
Satisfaction with salary		○		
Enough learning opportunities available			○	○
Clinic promotes professional growth			○	
Positive working environment				○
Regularly paid on time		○	●	●

^a^Most items were on a response scale of 0-3 (“not at all” to “a lot”)

^b^Binary 0/1 (“no”/”yes”) item

**Table 3:** Barriers to mental health service implementation reported across stakeholder levels

	Examples	Clients *(n *= *62)* N	Providers *(n *= *26)* N	Staff *(n *= *40)* N	Directors *(n *= *12)* N	Total *(N *= *140)* N
***Cultural barriers***						
Stigma	I am shy, people are very ruthless and make fun of mental illness (C) In our community, whoever suffers from mental illnesses is called crazy (S)	34	12	24	3	73
Beliefs, traditions	As long as that old belief circulate among us, we cannot make it feasible (P) Circulated customs, culture and tradition in the society make most challenges for the programme (S)	11	13	20	8	52
Predominance of traditional medicine	People believe more in sheik and mullah (C) There are people who believe in religious places to receive treatment and this makes it less acceptable (P)	9	8	9	2	28
***Lack of organizational resources***						
Center is too crowded, small space	Crowdedness of the place is an obstacle (C) Work condition in such a small hospital is very difficult (D)	14	17	22	6	59
Lack of designated/private place	Because of lack of place we would go to the courtyard (C) Lack of private place is a problem (S)	12	18	14	7	50
Lack of equipment/infrastructure	We have no access to computers to record files for patients (P) Psychotherapists do not have methods of communication with patients (S)	4	18	8	3	34
Center lacks financial resources	Lack of required budget (P) Financial obstacles is the most important (D)	1	8	17	6	32
Poor reputation of center	People have bad opinion and do not think it is good (C) They have little treatment and the doctors are not smart (C)	11	–	–	–	11
***Environmental challenges***						
Distance to facility	The place is far for patient to access (P) Most of the patients are poor and they cannot afford to come from far (S)	26	14	26	6	71
***Staffing issues***						
Too few staff/lack of time	We do not have sufficient employee for this service (S) Time allocated for seeing patients is short, mentally ill patients need more time (S) The therapist is very tired with patients (D)	6	6	19	5	36
Lack of therapists of same gender for clients	Sometimes patients [are] embarrassed talking to female therapist (P) I was shy to talk about everything to my CMHW because he was a male (C)	8	16	7	3	34
Lack of specialists	Shortage of psychotherapist makes problem (P) Lack of specialized psychiatrist is problem (S)	1	10	12	7	29
Lack of skilled or adequately trained staff	Lack of trained staff (P) This psychotherapist has received only 10 days training it is too early to become a psychotherapist (S)	–	5	7	5	17
***Program characteristics***						
Length of program	Duration of sessions is long (P) If allocated time is too long, it will make patients bored and it is less appropriate (S)	8	15	14	1	38
Low client adherence, drop-out	Patients are often find[ing] it difficult to continue receiving treatment (P) If patient does not apply recommendation and therapy perfectly, it will not be appropriate (S)	–	6	6	4	15
Inappropriate for context/needs	The religious aspect has not been mentioned in this program, which is the most important thing for treatment (C) This programme cannot be applied in this hospital (D)	9	–	–	2	11
No drugs	People are used to take drugs which means people are not calm to accept this treatment (S) People mostly believe in drugs than without it (D)	1	–	7	1	8
Program is new, untrusted	It is new, therefore some people do not trust it (P) Not trusting psychotherapist by the people, make[s] problem (S)	–	4	5	–	8
Dislike memory recall/talk therapy	The treatment is talk and I forget talk (what is said) (C) I do not like the painful memories of this program (C)	6	–	–	–	6
***Client logistical issues***						
Lack of transportation	[No] provisions [for] transportation methods for patients makes it not continue (P) Transportation for patients is [a] challenge (S)	13	3	7	–	22
Financial difficulties	From financial aspect, some people would like to come but they do not have access to it (C) There are patients who suffer economical problems and may not be able to come for treatment all the time (S)	5	5	4	1	15
Work interference	My problem is my work is daily. When I come here I lose it (daily wages) (C) When I ask for leave, I am confronted with many questions that I do not like (C)	13	–	–	–	13
Childcare issues	My problem is I have a child and do not know where to take him/her [when at treatment] (C) Because I have children and cannot leave them for a long time, that is my problem (C)	10	–	–	–	10
Home duties	And sometimes I was not able to apply the instructions because of domestic daily work (C) Illiterate people can’t apply this program because they miss their home duties (P)	2	4	2	–	8
***Low mental health literacy***						
Public unaware of service	It is a hidden thing, nobody knows this treatment is available (C) Lack of people[s] awareness to apply the programme is a problem (S)	12	5	19	5	41
Low education	Lack of people’s awareness (education) makes them visit the center with fear (C) [There is a] lack of awareness about mental health problems (P) People[s’] education about mental illnesses is not at required level (D)	4	11	6	4	25
***Lack of support for the program***						
Potential lack of external support	If international cooperation stops, it will be less sustainable (P) If international support stopped it will hard on the government to handle it (S)	–	18	18	1	36
Lack of government support	Government and concerned authorities do not handle the project as their own (P) The government is not taking this field into consideration, it does not take it seriously (S)	1	6	24	2	34
Lack of support from colleagues	Doctors do not help us, they do not refer patients (P) There are no harmony in our works (S)	–	4	15	–	20
Lack of organizational support	[There is a] lack of supervision (P) Non-cooperative director is a challenge (S)	–	4	2	1	8
***Lack of support for clients***						
Unsupportive family	Patients are not cooperated by their families (P) Sometimes my man (husband) is an obstacle saying do not go to the hospital (C)	27	9	16	4	56
Unsupportive friends/other supports	My friends say this treatment is ‘not serious’ (C) I was afraid my friends would look at me with a bad eye (think I am doing something bad) (C)	20	–	–	9	20

“–” indicates response was not provided at that stakeholder level

**Table 4:** Facilitators to mental health service implementation reported across stakeholder levels

	Examples	Clients *(n *= *62)* N	Providers *(n *= *26)* N	Staff *(n *= *40)* N	Directors *(n *= *12)* N	Total *(N *= *140)* N
***Positive perceptions of program***						
Program is good/people like it	I believe it is the best psychotherapy (P) People talk good about this therapy (C)	58	26	40	11	137
Program is acceptable/agrees with values	This therapy suits my values, otherwise I could not come (C) It is accepted by [when] explaining the treatment (P)	51	26	33	6	116
Program is suitable/appropriate	It suits my needs (C) This treatment is suitable and blessed (D)	47	26	29	11	113
Program is feasible	It is feasible and requires no changes (P) It is feasible with the current capacities (D)	–	18	27	10	55
Program is accessible/is for everyone	Everyone and [every] section of the community can have access to it (C)	7	–	–	–	7
***Program impacts***						
Is useful/effective	I had the feeling that I was getting better session after session (C) We were able to treat most of our patients’ problems (P) Patients get rid of psychological stress and it is useful for the community (D) The good thing is that patients recover to enjoy better normal health status (S)	58	18	24	5	106
Increased knowledge/MH literacy	I learned from it that not only I am ill (C) People’s awareness about mental health sicknesses increased (D)	9	–	15	4	28
No harmful impact	It does not have any bad effect for me (C)	27	–	–	–	27
Empowers clients	It enables patients to depend upon oneself (P) It teaches people better know their problems and have them solved by themselves (S) I have learned a lot from this treatment that I can control my mental issues (C)	14	5	2	–	21
Prevents suicide	Previously I had suicide idea but now it is no longer in my mind (C) This treatment helps decrease the level of suicide and self-burning (P)	9	1	5	–	15
Gives security, comfort	I had the feeling that I become comfortable when I come here (C) I felt secure (C)	15	–	–	–	15
Gives hope	I have regained hope for life (C) This mental health section gave hope to people, influenced them and people took benefit (S)	5	–	3	–	8
***Program attributes***						
Free of drugs	My problem was solved without taking a single pill (C) This treatment is better and more effective than drugs (S)	34	9	18	5	66
No cost	The treatment was free of charge (C) If treatment is free of charge, poor people will take benefit from the program (S)	29	13	14	4	60
Talk-based treatment	I could talk out what was in my heart (C) I needed someone to listen to me (C)	36	–	–	–	35
Simple/easy to follow	The home exercises made it easy (C) We feel the programme is not difficult, patients can apply there, therefore, it is feasible (P)	16	3	4	–	22
Flexible scheduling	They set the appointment according to my time (C)	16	–	–	–	15
Protects confidentiality	There was no name recording (C) Patients’ private data kept confidential by us (S)	12	1	2	1	15
Providers receive training	We received good trainings (P) In the university, I did not know anything, but here I learned (P)	–	13	–	–	13
Step-by-step/session-based	Weekly you sit with a CMHW for an hour or two; possibly you could not do so with someone else (C) This program is step-by-step that is why it will have effect on the patient (C)	7	–	–	–	7
***Provider attributes***						
Caring/respectful	There is someone who listens to you and trusts your decisions (C) We are always ready for any patient without difference (P) In this treatment, patients are respected and listened to (S)	54	6	12	–	73
Providers are capable/specialized	The CHHW was there ready at the exact time (C) There are capable psychotherapists (D)	13	8	11	3	35
Eager/motivated providers	As a psychotherapist, I continue and never quit (P) Staff eagerness and loyalty for the work (D)	–	10	–	3	13
***Service environment***						
Good facilities (large, quiet, clean)	The place was quiet (C) Our building is new and large (D)	35	7	17	3	62
Center has good reputation	The say good things about the reputation of the health center (C) The structure of the program is robust (C)	41	–	–	–	41
Separate space/MH section	There is a private place where I can talk (C) I have my own room (P)	10	4	–	3	18
Coffee/tea provided	They bring us water, tea and sweets (C)	12	–	–	–	13
Conveniently located	The place was very near for me (C) In terms of location, our hospital is suitably located (P)	8	1	1	–	10
***Positive work environment***						
Good cooperation among staff	They show readiness for giving their places whenever there is patients or when I ask them (P) Staff are very cooperative to the programme (S) Employees enjoy a good cooperation between them (D)	–	25	37	12	74
Supportive leadership	Supervision is at a very good level (S) The director facilitating a lot of things for us (P)	1	15	28	4	48
***Client attributes***						
Supportive friends/family	My mother told me many times to use this treatment to get better and this made me continue (C) My friends were happy that I came here (C)	53	–	–	–	53
Autonomy for treatment decisions	I come to this treatment based on my decision (C)	50	–	–	–	50
Able to arrange work/schedule	I arrange my work ahead of time (C) In my job, they give me permission [to leave] anytime I want (C)	21	–	–	–	21
Trust for the program/CMHW	The CMHW can be trusted so you talk about your problem with them (C) Patients tell psychotherapists their stories because they trust them (S)	10	4	5	–	20
Motivation/recognized need	I needed this treatment very much (C) I wanted to get rid of my problem (C)	19	–	–	–	20
Sufficient time available	I have time to come here (C)	12	–	–	–	13
Childcare available	I take my children to my neighbors (C)	11	–	–	–	11
***Adequate program resources***						
Financial/material support for clients and program	Paying the transport cost made it easy (C) Help from ‘material’ (Financial) aspect (C)	12	1	4	–	17
Provision of incomes for staff	Provision of incomes make it suitable to continue (S)	–	–	13	–	13
Adequate staffing/staff time	I allocate much time for patients (P) In the past, we suffered lack of doctors and social workers but now we have psychotherapist (S)	–	6	3	–	8

“–” indicates response was not provided at that stakeholder level

**Table 5:** Suggestions to improve mental health service delivery reported across stakeholder levels

	Examples	Clients *(n *= *62)* N	Providers *(n *= *26)* N	Staff *(n *= *40)* N	Directors *(n *= *12)* N	Total *(N *= *140)* N
***Change/improve facilities***						
Designated space for services	A private place should be allocated for mental health sections (S) A special (private) place be allocated for the CMHW (C)	16	13	33	9	71
Larger center	A larger mental health hospital should be available (P) We need a large hospital to be built for us (D)	6	5	3	2	15
Beautify facilities	The place [should] be in a garden surrounded by green areas (C) A library to be available in the center (C)	5	–	1	–	6
***Staffing changes***						
Increased specialist/trained staff	More expert psychotherapist and staff should be employed (S) Provision of smart and trained psychotherapist (D)	9	16	25	3	53
Increase staff numbers	More staff should be employed in this centre (S) Provide better number of doctors and staff (D)	3	4	21	8	36
Providers of matched gender	Both gender staff should be available in mental health section (S) More CMHW be available of both genders (C)	6	8	11	4	29
Protected time for providing services	No other jobs imposed on psychotherapists but the service only (P) Staff should carry out only this task in the hospital, not do any other tasks (S)	–	6	3	–	8
***Raise awareness***						
Mental health literacy	Media methods like TV, radio and newspapers should play their role on circulating awareness on mental health and this programme as well (P) To publicize more information about mental illnesses (D)	–	19	29	10	57
Educate about program	This treatment become known through media (C) This service should be introduced through media methods, symposiums and seminars (S)	9	14	8	2	34
***Increase support for program***						
Increase government support	Government and concerned authorities should take mental health more seriously (P) The government have to have a special plan for paying more attention to this program (C)	3	23	29	6	62
Financial/material support	Government and ministry to provide financial and moral support (P) If the government provide transportation means it will make the programme continue (S)	–	13	24	5	42
Formal recognition/integration	Government should recognize it formally (P) This programme to be included within ministry structure (D)	–	13	17	5	35
***Expand services***						
To all regions/centers	Now it is only available in one area I wish it was available in districts and sub-districts (C) More centres should be established for those who live far away (S)	19	6	17	1	43
Mobile teams	A mobile team to be available to visit remote areas (P) Mobile psychotherapist teams should visit patients in their homes (S)	2	3	13	3	21
Embed in schools/offices	Psychotherapist to be employed in schools (P) If they put CMHW in the schools it would make it easy (C)	1	1	3	1	6
***Program changes***						
Involve family	Family of patient should be talked with to support and cooperate the patient (S) If possible, patients’ families to participate in this treatment (P)	–	5	5	–	10
Adapt for illiterate clients	It is better to make a CD version for illiterate people (C) I prefer that the program use colors for illiterate people; for example, for something bad use a black color (C)	9	–	–	–	8
Treat patients carefully/with respect	Patients should be treated with respect (S)	–	–	8	1	8
Decrease sessions/time	Lessening the sessions (P) The sessions be reduced because it is difficult that the patient comes every week (C)	5	2	1	–	8
Encourage client adherence	It should be clear for the patient that the programme takes long time (S) In the beginning the programme should be explained for the patient (S)	–	–	6	–	6
***Financial supports***						
Allocate budget/resources for program	Budgets should be allocated for the programme (S) Money, staff, place, required equipment and transportation methods make continuity (D)	–	19	20	7	46
Accommodations for staff	Transportation and communication methods should provide for psychotherapists (S)	–	–	9	–	8
Financial support	Attempt to provide financial facilitation for patients (P) Financial support should be provided for patients (S)	4	6	5	4	20
Transportation support	Provide transportation fees for patients (P) It is necessary that means of transport be arranged for patients (C)	5	5	8	1	20
***Training***						
Provide trainings on the intervention	CETA to be inserted into college studies (P) More training courses should be arranged for the staff (S)	3	14	13	2	32

“–”indicates response was not provided at that stakeholder level

#### Perceived benefit from the program

A majority of all stakeholders (*n *= 106 overall; 58 clients, 18 providers, 24 non-MH staff, and 5 directors) reported perceptions that the program was useful or effective. Clients described specific impacts other than expected primary outcomes, including *emotional improvements* (*n* = 37; decreased anger and improved anger management, decreased feelings of depression, sadness, and anxiety); *improved or changed thinking* (*n* = 34); *physical improvements* (*n* = 24; improvements in appetite, sleep, pain, restlessness, energy); *social improvements* (*n* = 19; regained interest in relationships, decreased isolation); and *improved daily functioning* (*n* = 6; e.g. job performance).

Other impacts mentioned across stakeholders were: increased *awareness, knowledge and mental health literacy* (*n* = 28 overall, including 15 non-MH staff), *empowerment* of clients (*n* = 21 overall, including 14 clients), *reducing thoughts of self-harm* (*n* = 15 overall, including 9 clients and 5 non-MH staff), and offering a *sense of* *security and/or comfort* for the client (*n* = 15 clients). A question probing about potential harms from the treatment did not identify any prominent themes of harm.

Quantitative findings were similar; questions were posed to all four stakeholder groups assessing perceptions that the treatment was effective, helpful to people in the community, appropriate to the culture, and a fit to the presenting mental health problems. Clients were also asked about their overall satisfaction with the program and whether they would refer others to the program. All of these items received high positive endorsement with mean scores above 2.0.

#### The critical role of family and friends

Most clients (*n* = 37) and *n* = 30 other stakeholders reported *lack of support* from family and friends as barriers to care; for example, by perpetuating stigma, de-motivating clients, directly prohibiting participation, or not providing practical support (childcare, household help). Thirteen clients said that their spouse or family members were unaware that they had accessed treatment, saying, “my home do[es] not know about my illness, that is why I come by night,” or “my husband and children know I come here but they are unaware that I get psychological treatment”. *Having* such supports (e.g. emotional support and encouragement, transportation help, joining them in therapy, assisting with domestic duties) was reported as a significant facilitator by clients (*n* = 53). Often, adherence to the program appeared to be contingent on social support, family’s acceptance and acknowledgement of the client’s condition and their treatment. Yet only a minority of providers (*n* = 5) and staff (*n* = 5) suggested involving family in treatment.

Quantitative items around client autonomy to seek treatment presented a mixed picture of decision-making power and influence. Clients reported a high amount of agreement with statements that they feel able to make decisions about their mental health care (*M *= 2.81, *SD *= .43) and that they are the person in their family who decides whether they get mental health care (*M *= 2.57, *SD *= .79). Yet a reverse-coded item asking the extent to which *other* people in their family make these decisions for them was also highly endorsed (*M *= 2.02, *SD *= 1.20). All stakeholder groups identified low service accessibility for women as particularly problematic (clients: *M *= 1.94, *SD *= .81; providers: *M *= 1.72, *SD *= .79; non-MH staff: *M *= 1.29, *SD *=* .80*; directors: *M *= 1.75, *SD *=* .62*), which may reflect a shared decision-making process particularly impacting women.

#### Need for greater organizational support for the program

Although most employees reported supportive working relationships (including 25 providers and 37 non-MH staff), concerns about organizational climate issues were also raised; particularly a lack of support from colleagues (e.g. negative attitudes, limited teamwork). Fifteen non-MH staff (as well as four providers) mentioned these issues, describing problems with getting support and referrals, such as “doctors do not consider [the mental health program] as their duty and this is the challenge”, “there are some staff who do not believe in [the mental health treatment] and do not like it”, and, “some of my colleagues ridicule [the program]”. Directors appeared to be unaware of these concerns, as all reported good cooperation and none mentioned lack of support from colleagues.

In the quantitative data, issues of being overworked (a reverse-coded item) and not paid on time, respectively, were identified among providers (*M *= 1.19, *SD *= *1.02; M *= 1.73, *SD *= *1.00)*, non-MH staff (*M *= 2.08, *SD *= *.97; M *= 0.91, *SD *= *1.06)*, and directors (*M *= 2.08, *SD *= *1.31; M *= 1.00, *SD *= *1.10)*. Providers also reported some dissatisfaction with their salary (*M *= 1.85, *SD *=* 1.05*), while non-MH staff reported a lack of learning opportunities (*M *= 1.74, *SD *=* 1.12*) or promotion of professional growth (*M *= 1.81, *SD *=* .92*). Regarding other issues impacting feasibility, non-MH staff and directors identified challenges related to budget (*M *= .53, *SD *=* .80; M *= .55, *SD *=* .82*), other resources (*M *= .91, *SD *= *.95; M *= 1.18, *SD *= *.87*) and overall feasibility of mental health service integration (*M *= 1.61, *SD *=* 1.06; M *= 1.75, *SD *=* .87*). Providers, who were asked a more specific set of questions about service delivery, indicated poor access to technology needed to provide services (*M *= 1.44, *SD *=* 1.40*), but less of a problem getting access to other necessary equipment such as pens and paper (*M *= 2.36, *SD *=* .95*).

#### Program length could be a challenge

Thirty-eight stakeholders raised concerns about program length; this theme was markedly higher among providers (*n* = 15), who were most knowledgeable about reasons for client dropout, as well as non-MH staff (*n* = 14). Providers (*n* = 6), non-MH staff (*n* = 6), and directors (*n* = 4) also described related issues of low client adherence and dropout (e.g. “parents are often find[ing] it difficult to continue receiving treatment” presumably due to childcare, transport, and other household obligations).

Clients responded positively to a quantitative item assessing the extent to which they had the necessary *time* to attend sessions (*M *= 2.45, *SD *= .77), but another item assessing the difficulty they had attending weekly treatment sessions for 8–12 weeks was identified as a potential barrier (*M *= 1.14, *SD *= 1.10 on a reverse-scored item). Although clients did report that the services were accessible for them (*M *= 2.49, *SD *= .66), all stakeholder groups identified issues of low service accessibility among groups in the community overall (Table 2).

#### Lack of dedicated mental health space

Resource challenges as barriers were not unexpected, yet issues of lack of space were particularly pressing. Crowded centers were reported by nearly a quarter of clients (*n* = 14) and over half of all other stakeholders (17 providers, 22 non-MH staff, and 6 directors), with similar numbers reporting lack of private or designated mental health space (12 clients, 18 providers, 14 non-MH staff, and 7 directors). Quantitative data also identified lack of access to private space for client meetings as a perceived barrier by providers (*M *=1.65, *SD *= 1.30), non-MH staff (*M *=1.09, *SD *= 1.20) and directors (*M *=1.33, *SD *= 1.15). Qualitative descriptions of conducting sessions in a courtyard or staff giving up their offices for sessions highlight the impact of these challenges. Half of all stakeholders (16 clients, 13 providers, 33 non-MH staff and 9 directors) recommended that a designated space be made available for services. In most cases, this appeared to refer to space within primary care areas as only 15 stakeholders specifically mentioned needing a larger mental health hospital.

#### Insufficient staffing

Nearly half of non-MH staff (*n* = 19) and directors (*n* = 5) described insufficient staffing to provide mental health services in primary care; in contrast, only six providers mentioned the same. This same trend is observed in the quantitative data, where non-MH staff and directors primarily identified issues of sufficiency of counselors (non-MH staff: *M *= 1.53, *SD *=* .93;* directors: *M *= 1.83, *SD *=.94) and counselor time (non-MH staff: *M *= 1.86, *SD *=* 1.06;* directors: *M *= 1.92, *SD *=1.00), whereas counselors reported having enough time to provide mental health services (*M *= 2.35, *SD *= .69). All three of these stakeholder groups also described a lack of providers of matched gender for clients (*n* = 26), lack of sufficient specialist providers such as psychiatrists (when referrals were needed) (*n* = 29) and a shortage of other skilled or adequately trained staff (*n* = 17). In comparison, only a small minority of clients mentioned staffing concerns (6 relating to shortages, 8 relating to matched gender concerns).

*Perceived lack of government support for the program* was a prominent concern in the qualitative data, primarily among non-MH staff (*n* = 24). Some stakeholders expressed concerns that, without international support, the program would not continue (18 providers, 18 non-MH staff). In the quantitative data, these groups indicated a lack of government support for the program (providers: *M *= 1.27, *SD *= 1.03, non-MH staff: *M *= 1.22, *SD *=* .75*; directors: *M *= 1.64, *SD *= *.67*), although they appeared to remain hopeful that the program would continue after external support ended (providers: *M *= 2.31, *SD *= 1.09, non-MH staff: *M *= 2.60, *SD *=* 1.17*; directors: *M *= 2.92, *SD *=* 1.0*). Qualitative suggestions highlighted a need for greater financial support (13 providers, 24 non-MH staff, and 5 directors), a desire for the government to formally recognize and integrate the service (13 providers, 17 non-MH staff, and 5 directors), as well as general statements that the government should take mental health concerns seriously (23 providers, 29 non-MH staff, and 6 directors).

Other findings reflect challenges that are well documented in existing literature [28–32] and so are described here only briefly. These included barriers such as (1) stigma, beliefs or traditions, and predominance of traditional medicine (i.e. seeking treatment outside the health system); (2) gender mismatch between client and provider; (3) low mental health literacy; (4) lack of budget and other resources and/or dedication to mental health; (5) distance and lack of services in rural areas; and (6) client logistical issues (e.g., transportation, finances, other responsibilities, childcare). These barriers were consistent across both the qualitative and quantitative data. Recommendations included television or radio campaigns, service expansion (to rural areas, or mobile clinics), improving facilities and providing additional CETA trainings. Recognized facilitators included: (1) general positive perceptions of the program such that it was a “good” fit with values or culture and was suitable or appropriate; (2) providers who were perceived as respectful, caring and capable; (3) service environments that had good facilities (large, quiet, clean) or a convenient location; (4) work environment characteristics such as good cooperation, supportive leadership; and having adequate program resources; and (5) the program was free of cost and medications, simple to follow, had a good reputation and offered flexible scheduling. Some clients also described support provided by the clinic (e.g. transportation funds) that enabled them to attend treatment.

## Discussion

This mixed methods study is unique in its inclusion of four distinct stakeholder perspectives on integration of evidence-based mental health services in 12 primary care clinics in Northern Iraq. The ultimate aim of this study was to gain information on building sustainable, integrated mental health programs, requiring that we obtain input from a wide range of stakeholders. In particular, when integrating mental health into non-mental health programs, it became clear that the views of non-mental health stakeholders, such as others in the organization not engaged in the delivery of mental health services, were critical to understand. In particular, comparing perspectives across mental health providers, non-MH staff and directors allowed us to identify both issues of broad consensus (e.g. generally positive perceptions of the program’s acceptability and effectiveness, concerns about lack of government support, concerns about space), as well as critical areas of divergence in perspectives that illustrate challenges in service integration. These findings, some of which were quite unexpected, highlight the importance that service providers carry out these sorts of stakeholder engagement efforts when seeking to integrate services in order to develop an integrated service that is feasibly implemented and sustainable [8, 33].

Non-MH staff—who were generally supportive and reported an overall positive working environment—described an added burden of the program in terms of time, task allocation, and space/resource constraints that disproportionally impacted them. Some directors appeared to have limited awareness of these issues, highlighting a common disconnect between management and staff which is a well-recognized focus in implementation literature [34–36]. Providers, who also appeared to have a generally positive view of service integration, did not appear to fully understand the potential added burden on their colleagues. On the other hand, provider perspectives were key to understand probable challenges faced by clients who did not complete services and whose voices were therefore underrepresented in our study; it was in these provider interviews that issues of acceptability and feasibility arose, particularly in terms of gender issues and program length. From those clients who we *were* able to interview, most of whom successfully completed the program, feedback suggested that the program was viewed quite favorably, with primary concerns being about low accessibility, logistical challenges to attending sessions, and need for family support. The value of each of these perspectives supports including multiple stakeholder groups in future research to evaluate integration efforts.

Many of the themes we identified are interrelated and, taken together, provide valuable lessons for integration of mental health into non-mental health sectors. For example, CETA, like many other outpatient mental health programs, is an 8–12 week, talk-based intervention; barriers included providers in primary care settings ability to deliver multiple sessions, space and resources at non-mental health service centers, and client’s lack of support from friends and family. This study suggests that integration in primary care settings with health workers presents challenges of balancing tasks and that multiple visits as a standard treatment approach could be foreign and difficult to fit in with other tasks. In this setting, most of the community health workers were used to job tasks that were one-off such as vaccinations or running a health test. This suggests that future projects may benefit from assessing job descriptions of potential mental health providers, and consider utilizing non-mental health settings but dedicated mental health providers that do not need to balance their tasks. Specifically, we recommend clearer delineation between tasks, and either designating identified staff as mental health providers full time, or otherwise designating certain days or times per week that are set and therefore enable planned shifts in other human resources to accommodate their time (see, for example: [37]). Integration within these primary health clinics also raised significant issues around resource allocation for mental health versus non-mental health services—and likely interacts with the perceived lack of organizational support some providers reported. This is consistent with findings from elsewhere that report similar challenges and suggest acceptability of task-sharing in mental health services is contingent on availability of increases in human and other resources, supervision supports, training, and compensation [38, 39]. It will be helpful in the future to discuss resource allocation when integrating mental health with non-mental health programming, including specific plans for designated spaces for service provision, and include communication of this plan across all stakeholder levels. Some clients reported a need to attend services in secret. It is possible that in these cases, integrating mental health services within primary care facilities in order to ‘mask’ participation may facilitate these clients’ ability to receive mental health care. This is consistent with findings from the US that patients endorsing higher preference for integrated primary care also report higher stigma [40]. Likewise, arguments in support of integrated models highlight the potential for increased access, patient-centered care, and reduced stigma [8].

CETA service implementation took place in collaboration with the MoH-run clinics, yet there remained significant lack of general awareness regarding the intervention reported by all stakeholders and concern among provider, non-MH staff and directors about a lack of support and formal recognition from the government. While non-MH staff and directors were largely supportive of the program, providers reported that some colleagues held negative views of the program or of mental health more generally suggests that mental health stigma also exists in the healthcare environment and among healthcare providers, which is a challenge not unique to LMIC [41, 42]. Some of the suggestions from staff about modifying the program—for example, to include psychoeducation and engage family members when feasible—describe existing program elements, reflecting a potential lack of knowledge about treatment components or that the current content is insufficient. While there was meant to be a basic orientation among clinic staff and directors about the intervention, in future initiatives a more intentional and multi-tiered information and stakeholder engagement campaign or more collaborative care model may be warranted to overcome these challenges and streamline performance of the health system as a whole [43, 44]. The lack of general awareness and formal recognition by the government are both likely to be significant barriers to sustainability; parallel efforts to expand and professionalize mental health service delivery, such as by integrating mental health care into university training programs are also recommended [45, 46]. Other studies utilizing similar task-shifting approaches, noted similar concerns of sustainability and good governance [47], as well as risk that a lack of publicity related to task-shifting programs could result in misconceptions within the health system [48]. Use of media and community outreach activities were frequently suggested by stakeholders as a promising approach to increase mental health literacy more generally as well as knowledge about and visibility of the program.

The integration of mental health services into primary healthcare clearly presented human resource challenges. Providers ranged from those who were dedicated mental health providers to those who took on a mental health role in addition to continuing other types of duties (nursing, pharmacy staff, etc.). Although providers’ workloads were meant to shift to accommodate their new roles, there were issues with both number of providers and availability of sufficient time for service provision, as well as concerns about salary commensurate with the current workload, reflecting similar concerns reported elsewhere [39, 48]. Task-shifting likely also required other non-MH staff to take on more work, perhaps contributing to their more critical view of the service. On the other hand, support was described as present from some staff; for example, in some cases staff gave up their own office to the CMHW to allow for privacy for a client. The necessity for social support and supervision within the workforce has been noted for mental health task sharing programs [39, 49]. There was recognition from all levels for the need to adjust, which may suggest that the communication and problem-solving around integration needs more focused attention. A 2013 systematic review highlighted the need for regular communication within the workforce, noting that critical views of co-workers regarding task-shifting programs was often due to lack of awareness or unclear role delegations [50]. While ongoing supervision is already recommended for building clinical competencies [13], our experience supports the need for additional supervision focused on these organizational aspects of service integration.

This implementation period coincided with increased instability in the Kurdistan region due to an economic crisis and escalating conflict with the Islamic State (ISIS). Two providers stopped providing CETA services so that they could go support medical facilities serving wounded soldiers. Gas prices escalated sharply, increasing the burden to clients attempting to seek treatment. Clients in some areas may have been less likely to continue treatment due to security concerns. Additionally, the holy and fasting month of Ramadan fell in July, where daily high temperatures regularly exceed 40 °C, leading to a decreased in clinical activity. These are all factors that may have exacerbated typical barriers to service uptake and delivery, and may have also influenced study findings. For example, given the MoH involvement in planning and supporting this initiative, the overwhelming lack of stakeholder confidence in MoH support was unexpected, but may reflect shifting MoH priorities over this timeframe to respond to the unfolding events. Historic and current political conflicts have been recognized as eroding stakeholder confidence and uptake of mental health initiatives elsewhere [38].

## Strengths and limitations

Beyond five clients, we were unsuccessful in engaging with potential clients who chose not to receive services; theirs’ is a critical and missing voice in this study. Even among those who initiated, our use of a convenience sample resulted in a client sample that is biased toward those who adhered to, and mostly completed, services. Convenience sampling at the non-MH staff level may have also introduced some bias, and is a limitation. We were, however, able to incorporate the perspectives of all providers and of two additional types of stakeholders, non-MH staff and directors, who often are not represented. Providers were able to share insights about why clients dropped out of treatment, but their insights cannot be presumed to extend to those potential clients who never initiated services.

This study also leveraged quantitative data on implementation science instruments which, to date were translated, back translated and examined for psychometric properties, yet not formally validated. To accommodate this limitation, we conducted extensive qualitative interviews, which ultimately strengthened our ability to provide a mixed-methods analysis of results. We also analyzed quantitative data at the individual item level rather than using scale scores, which reduces the psychometric complexity of the data. The clear consistency between qualitative and quantitative responses supports the integrity of the findings presented here. Perhaps due to the volume, our qualitative interviews often did not probe enough to answer all the questions that the current findings raise, leaving hints but many remaining knowledge gaps ripe for further study.

Finally, this study was only conducted among a sample of clinics in one region of Iraq, and as noted above, during a period of time in which this region was exposed to a rapidly changing security and political context. Given these contextual challenges, findings should not be presumed to generalize to other parts of Iraq or to other countries.

## Conclusion

Using a mixed-methods approach to study the integration of an evidence-based, mental health approach (CETA) into primary care clinics in Northern Iraq, we found that perceptions of the program were generally positive, but that challenges remain in terms of how best to integrate the program into primary care. Numerous insightful barriers, facilitators and suggestions to implementation were provided across the four stakeholder levels. Implementation evaluations in LMIC have rarely included the perspectives of non-mental health staff working in the clinics in which services were integrated; our study illustrates the benefit of taking a broader approach. In this study, providers reported generally positive integration efforts but some unsupportive colleagues, whereas non-MH staff—although largely supportive of the program—did report negative impacts on their working environment beyond what providers seemed to recognize. Directors often appeared unaware of these tensions. Clients, however, reported many positive benefits of the program, some beyond those the program was designed to address. This type of study will be increasingly necessary to overcome challenges that have been encountered in the integration of mental health services into other service platforms in LMIC and start to design implementation strategies to better roll-out integration efforts [8, 51, 52].

## Supplementary information

**Additional file 1.** Sample quantitative questionnaire.

**Additional file 2.** Sample qualitative interview guide.

## Data Availability

The datasets used and analyzed during the current study are available from the corresponding author on reasonable request.
