# Supplementary material for: Stakeholder perspectives on integration of mental health services into primary care: a mixed methods study in Northern Iraq
Source: Int J Ment Health Syst. 2019 Dec 28;13:75. doi: 10.1186/s13033-019-0330-7 (PMC6935235; doi:10.1186/s13033-019-0330-7)
Supplement: Supplementary file 1 — Additional file 1. Sample quantitative questionnaire. [file 13033_2019_330_MOESM1_ESM.pdf]

Organizational Questionnaire – TIME 1, 2 — **پرسیارنامەی رێکخراو– کات 1، 2**

To be completed by the interviewer or project director: — **دەبێت لەلایەن دیمانەساز یا بەرێوەبەری پرۆژەوە پربکرێتەوە:**

**Respondent ID #:**			کۆدی وەلامدەر
Today's date			بەرواری ئەمرۆ
Time-point	1<br>Baseline<br>یەکەم جار	2<br>follow-up<br>دوەم جار	کات
Interviewer ID #			کۆدی دیمانەساز

Instructions to interviewer: — **رێنمایی بۆ دیمانەساز**

Circle the answer for each question. If person refuses to answer a question, cross out whole row for that question with one line.

بازنەیەك بكێشە بەدەوری وەلامەكاندا.ئەگەر كەسەكە رازی نەبوو وەلامی پرسیارێك بداتەوە یان وەلا می پرسیاركی نەدەزانی ئەوا هێلێك بەسەر ئەو پرسیارەدا بهێنە.

1. Demographic Characteristics — **1-پێناسەی وەلامدەر**

1.1.	Record gender of respondent.	0<br>Male<br>نێر		1<br>Female<br>مێ		رەگەزی وەلامدەر؟	1،1
1.2.	What is the year of your birth?					مواليدت چەندە؟	1،2
1.3.	What is the highest level of education you have completed?	0<br>None<br>هیچ	1<br>Primary<br>سەرەتایی	2<br>Secondary<br>ناوەندی یان ئامادەیی	3<br>Institutional degree/ Certificate<br>بروانامە و پلەی پەیمانگا	5<br>Bachelor's or higher<br>بەکالۆریۆس یا بەرزتر	بەرزترین ئاستی خوێندنت چیە کە تەواوت کردوە؟	1،3

2. Adoption

2-پەیرەو کردن

	Question پرسیار	Not at all بە هیچ شێوەیەك	A little bit کەمێك	A moderate amount تا ڕادەیەك- مام ناوەند	A lot زۆر	Do not know نازانم	Question پرسیار	
2.1.	How much do you support implementing step-by-step psychotherapy services in the clinic where you work? (24)	0	1	2	3	9	تا چەندێك پاڵپشتی جیبەجێکردنی خزمەتگوزاری چارەسەری دەرونی هەنگاو بەهەنگاو دەکەیت لەو بنکە تەندروستییەی کاری تیادەکەیت؟. (24)	2،1
2.2	How much assistance could you provide, if needed, to integrate step-by-step psychotherapy into clinic services? (15)	0	1	2	3	9	تا چەندێك دەتوانیت یارمەتیدەربیت لەزیادکردنی چارەسەری دەرونی هەنگاو بەهەنگاو بۆ خزمەتگوزارییەکانی بنکە تەندروستییەکە؟ (15)	2،2
2.3.	To what extent would you refer patients with mental health problems to receive step-by-step psychotherapy services? (16)	0	1	2	3	9	تا چ رادەیەك ئەو نەخۆشانەی گرفتی تەندروستی دەرونیان هەیە ڕەوانەی خزمەتگوزاری چارەسەری هەنگاو بە هەنگاو دەکەیت؟ (16)	2،3

3. Acceptability **3- قبوڵكردن (پەسەندكردن)**

	Question<br>پرسيار	Not at all<br>بە هيچ شێوەيەك	A little bit<br>كەمێك	A moderate amount<br>تا ڕادەيەك- مام ناوەند	A lot<br>زۆر	Do not know<br>نازانم	Question<br>پرسيار	
3.1.	To what extent is step-by-step psychotherapy a priority for the government?(19)	0	1	2	3	9	تا چ رادەيەك چارەسەری دەرونی هەنگاو بەهەنگاو لای حكومەت ئەولەويەتی هەيە؟ (19)	3,1
3.2.	How much does your job negatively affect your family life? (40)	0	1	2	3	9	تا چەندێك ئيشەكەی تۆ كاريگەری خراپی لەسەر ژيانی خێزانيت هەيە؟ (40)	3,2
3.3.	How appropriate is step-by-step psychotherapy for your culture? (26)	0	1	2	3	9	تا چەندێك چارەسەری دەرونی هەنگاو بەهەنگاو گونجاوە بۆ كەلتوری تۆ؟ (26)	3,3
3.4.	To what extent is step-by-step psychotherapy a priority for the administration/leadership of the clinic? (27)	0	1	2	3	9	تا چ رادەيەك چارەسەری دەرونی هەنگاو بەهەنگاو ئەولەويەتی هەيە لە لای بەرێوەبەرايەتی بنكە تەندروستيەكە؟ (27)	3,4
3.5.	How acceptable do you believe step-by-step psychotherapy is to your health center? (28)	0	1	2	3	9	تا چەندێك پێت وايە چارەسەری دەرونی هەنگاو بەهەنگاو مەقبولە لە لايەن بنكە تەندروستيەكەت؟ (28)	3,5
3.6.	How acceptable do you believe step-by-step psychotherapy is to this community? (29)	0	1	2	3	9	تا چەندێك پێت وايە چارەسەری دەرونی هەنگاو بەهەنگاو لەم كۆمەڵگاكەيەدا مەقبولە؟ (29)	3,6
3.7.	To what extent does the step-by-step psychotherapy program fit with how this clinic functions/does things? (25)	0	1	2	3	9	تا چ رادەيەك پرۆگرامی چارەسەری دەرونی هەنگاو بەهەنگاو لەگەڵ چۆنيەتی كاركردنی ئەم بنكە تەندروستيەدا دەگونجێت؟ (25)	3,7

4. Appropriateness

4- گونجاوی

	Question پرسیار	Not at all به هیچ شێوەیەك	A little bit کەمێك	A moderate amount تا ڕادەیەك- مام ناوەند	A lot زۆر	Do not know نازانم	Question پرسیار	
4.1.	Given the other health problems people have, how important is it for the government to allocate resources (e.g. time, salary) to step-by-step psychotherapy? (1)	0	1	2	3	9	بەرەچاوکردنی پێداویستیە پێداویستیەکانی تری خەڵك، تا چەندێك گرنگە سەرچاوەکانی وەکو پارە و کات بۆ چارەسەری دەروونی هەنگاو بە هەنگاو لە لایەن حکومەتەوە دابین بکرێت؟ (1)	4،1
4.2.	To what extent does the step-by-step psychotherapy program fit with this clinic's priorities and goals? (3)	0	1	2	3	9	تا چ رادەیەك پرۆگرامی چارەسەری دەرونی هەنگاو بەهەنگاو لەگەڵ ئامانج و ئەولەویەتەکانی ئەم بنکە تەندروستیە دەگونجێت؟ (3)	4،2
4.3.	How appropriate is step-by-step psychotherapy for clients' mental health problems? (4)	0	1	2	3	9	تا چەندێك چارەسەری دەرونی هەنگاو بەهەنگاو بۆ گرفتی تەندروستی دەرونی نەخۆشەکان دەگونجێت؟ (4)	4،3
4.4.	How helpful do you believe step-by-step psychotherapy is for this clinic? (5)	0	1	2	3	9	تا چەندێك پێت وایە چارەسەری دەرونی هەنگاو بەهەنگاو بۆ ئەم بنکە تەندروستییە بەسودە؟ (5)	4،4
4.5.	How helpful do you believe step-by-step psychotherapy is for this country? (6)	0	1	2	3	9	تا چەندێك پێت وایە چارەسەری دەرونی هەنگاو بەهەنگاو بۆ ئەم ولاتە بەسودە؟ (6)	4،5
4.6.	How helpful do you believe step-by-step psychotherapy is for people in this community? (7)	0	1	2	3	9	تا چەندێك پێت وایە چارەسەری دەرونی هەنگاو بەهەنگاو بۆ خەڵکی ئەم کۆمەڵگایە بەسودە؟ (7)	4,6

4.7.	To what extent does providing step-by-step psychotherapy negatively affect provision of other services? (8)	0	1	2	3	9	تا چ رادەیەك پێشكەشكردنی چارەسەری دەرونی هەنگاو بەهەنگاو كاریگەری خراپ دەكاتە سەر پێشكەشكردنی خزمەتگوزارییەكانی تر؟ (8)	4,7

5. Autonomy

5- سەربەخۆیی

	Question<br>پرسیار	Not at all<br>به هیچ شێوەیەك	A little bit<br>كەمێك	A moderate amount<br>تا ڕادەیەك- مام ناوەند	A lot<br>زۆر	Do not know<br>نازانم	Question<br>پرسیار	
5.1.	How much does the clinic where you work trust your professional judgment? (22)	0	1	2	3	9	تا چەندێك ئەو بنكە تەندروستییەی ئیشی تێدا دەكەیت، متمانەیان بە بڕیارەكانت هەیە؟ (22)	5،1
5.2.	To what extent do you feel that you have an appropriate level of autonomy to make decisions in your work? (23)	0	1	2	3	9	تا چ رادەیك هەست دەكەیت كە ئاستێكی گونجاوی سەربەخۆییت هەیە بۆ ئەوەی بڕیار بدەیت لەكارەكەتدا؟ (23)	5،2
5.3.	How comfortable do you feel making suggestions to your boss/supervisor? (46)	0	1	2	3	9	تا چەندێك هەست دەكەیت دەتوانیت بە ئیسراحەت پێشنیاربكەیت بۆ بەڕێوەبەرێتی بنكە تەندروستییەكە؟(46)	5،3
5.4.	To what extent do you feel like you could make suggestions at work that might go against others' viewpoints? (47)	0	1	2	3	9	تا چ رادەیەك هەست دەكەیت دەتوانیت لەكارەكەتدا ئەو پێشنیارانە بخەیتە ڕوو كە لەگەڵ بۆچونی كەسانی تردا نین؟ (47)	5،4

5.5	Who has a role in making decisions at the clinic such as what the clinic is planning to do and how it will be carried out)? (circle all that apply) (50)	1 Providers چارەساز	2 Other clinical staff کارمەندی تری بنکە تەندروستییەکە	3 Administrative staff کارمەندانی کارگێری	4 Directors بەڕێوەبەرەکان	5 Others (specify) کەسانی تر (دیاری بکە)	لە شوێنی کارەکەت کێ دەوری هەیە لە بریاردانندا (بۆ نمونە بنکە تەندروستییەکە پلانی هەیە چی بکات، هەروەها چۆن ئەنجامی دەدات (بازنە بکێشە بەدەوری وەڵامە راستەکاندا) (50)	5،5

6. Step-by-step Psychotherapy Knowledge

6-زانینی چارەسەری دەرونی هەنگاو بەهەنگاو

	Question<br>پرسیار	Responses<br>وەڵامەکان			Question<br>پرسیار	
6.1.	Yes or no: Step-by-step psychotherapies are long term treatments that lasts a minimum of a year (51)	0<br>No<br>نەخێر	1<br>Yes<br>بەڵێ	9<br>Do not Know<br>نازانم	چارەسەری دەرونی هەنگاو بەهەنگاو درێژخایەنەو لانی کەم ساڵێك دەخایەنێت (51)	6,1
6.2.	Yes or no: Step-by-step psychotherapy is a treatment that is shown to be effective in Kurdistan for adult mental health problems (52)	0<br>No<br>نەخێر	1<br>Yes<br>بەڵێ	9<br>Do not Know<br>نازانم	بەڵێ، نەخێر: لە کوردستاندا، چارەسەری دەرونی هەنگاو بەهەنگاو بۆ گرفتەکانی تەندروستی دەرونی کەسانی پێگەیشتوو، کاریگەرە (52)	6,2،6
6.3.	Yes or no: Step-by-step psychotherapies focuses on thoughts, feelings and behaviors (54)	0<br>No<br>نەخێر	1<br>Yes<br>بەڵێ	9<br>Do not Know<br>نازانم	چارەسەری دەرونی هەنگاو بەهەنگاو تەرکیزدەکاتە سەر هزر (فکر) و هەست و رەفتار (54)	66,3
6.4.	Yes or no: Step-by-step psychotherapy is a talk-based counseling program.	0<br>No<br>نەخێر	1<br>Yes<br>بەڵێ	9<br>Do not Know<br>نازانم	چارەسەری دەرونی هەنگاو بەهەنگاو بەندە لەسەر ڕاوێژکاری گفتوگۆ.	6,4

7. Feasibility

7- عەمەلیبون

	Question<br>پرسیار	Not at all<br>بە هیچ شێوەیەك	A little bit<br>کەمێك	A moderate amount<br>تا ڕادەیەك- مام ناوەند	A lot<br>زۆر	Do not know<br>نازانم	Question<br>پرسیار	
7.1.	To what extent does the clinic have enough counselors to implement step-by-step psychotherapy? (32)	0	1	2	3	9	تا چ رادەیەك بنكە تەندروستییەكە چارەسازی پێویستی هەیە بۆ جێبەجێكردنی چارەسەری دەرونی هەنگاو بەهەنگاو؟ (32)	7،1
7.2.	To what extent does the clinic have enough counselor time to implement step-by-step psychotherapy?	0	1	2	3	9	تا چ رادەیەك چارەسازەكانی بنكە تەندروستیەكە كاتی تەواویان هەیە بۆ جێبەجێكردنی چارەسەری دەرونی هەنگاو بەهەنگاو؟	7,2
7.3.	To what extent does the clinic have sufficient budget to implement step-by-step psychotherapy? (61)	0	1	2	3	9	تا چ رادەیەك بنكە تەندروستییەكە بودجەی تەواوی هەیە بۆ جێبەجێكردنی چارەسەری دەرونی هەنگاو بەهەنگاو؟ (61)	7,3
7.4.	To what extent does the clinic have enough private space to implement step-by-step psychotherapy? (62)	0	1	2	3	9	تا چ رادەیەك بنكە تەندروستیەكە شوێنی تایبەتی هەیە بۆ جێبەجێكردنی چارەسەری دەرونی هەنگاو بەهەنگاو؟ (62)	7,4

	Question<br>پرسیار	Not at all<br>بە هیچ شێوەیەك	A little bit<br>کەمێك	A moderate amount<br>تا ڕادەیەك- مام ناوەند	A lot<br>زۆر	Do not know<br>نازانم	Question<br>پرسیار	
7.5.	To what extent does the clinic have enough other necessary resources to implement step-by-step psychotherapy? (Some examples could be support staff, administrative time, transportation money, or any other resources you might need.) (33)	0	1	2	3	9	تا چ رادەیەك بنكە تەندروستیەكە سەرچاوەی تەواوی تری بەدەستەوەیە بۆ جێبەجێكردنی چارەسەری دەرونی هەنگاو بەهەنگاو (بۆ نمونە، كارمەندی كارگێری، كات، هۆیەكانی گواستنەوە، پارە، یان هەر سەرچاوەیەكی تر كە پێویست بوو(؟ (33)	7,5
7.6.	How feasible is integrating step-by-step psychotherapy into primary health centers? (35)	0	1	2	3	9	تا چەندێك ئاوێتەكردنی (دمج)چارەسەری دەرونی هەنگاو بەهەنگاو لە بنكە تەندروستییەكاندا شتێكی عەمەلیە؟ (35)	7,6
7.7.	To what extent is staff turnover a problem here at this clinic? (36) (feas; POS)	0	1	2	3	9	تا چ رادەیەك جێگۆركێ و وازهێنانی كارمەند لەم بنكە تەندروستیەدا گرفتە (36)	7,7
7.8.	To what extent is the staff at this clinic paid on time consistently? (38) (feas; POS)	0	1	2	3	9	تا چ رادەیەك كارمەندانی ئەم بنكە تەندروستیە لە كاتی دیاریكراودا موچە وەردەگرن؟ (38)	7,8

8. Penetration

8- بڵاوبوونەوە

Question پرسیار		Not at all به هیچ شێوەیەك	A little bit کەمێك	A moderate amount تا ڕادەیەك- مام ناوەند	A lot زۆر	Do not know نازانم	Question پرسیار	
8.1.	How accessible is step-by-step psychotherapy for most people in the community?(55)	0	1	2	3	9	تا چەندێك چارەسەری دەرونی هەنگاو بە هەنگاو دەگاتە زۆربەی خەڵك لە کۆمەڵگادا؟ (55)	8،1
8.2.	How accessible is step-by-step psychotherapy for the poorest people in the community? (56)	0	1	2	3	9	تا چەندێك چارەسەری دەرونی هەنگاو بە هەنگاو دەگاتە هەژارترین خەڵك لە کۆمەڵگادا؟ (56)	8،2
8.3.	How accessible is step-by-step psychotherapy for women who need mental health services? (57)	0	1	2	3	9	تا چەندێك چارەسەری دەرونی هەنگاو بە هەنگاو دەگاتە ئەو ژنانەی پێویستیان بە خزمەتگوزاری تەندروستی دەرونی هەیە؟ (57)	8،3
8.4.	How accessible is step-by-step psychotherapy for men who need mental health services? (58)	0	1	2	3	9	تا چەندێك چارەسەری دەرونی هەنگاو بە هەنگاو دەگاتە ئەو ئەو پیاوانەی پێویستیان بە خزمەتگوزاری تەندروستی دەرونی هەیە؟ (58)	8،4
8.5.	Are there are groups in the community who cannot access step-by-step psychotherapy? (60)	0 No نەخێر		1 Yes بەڵێ		9 Do not Know نازانم	ئایا لە کۆمەڵگاکەدا هیچ گروپێك هەن کە دەستیان بە چارەسەری دەرونی هەنگاو بە هەنگاو ناگات؟ (60)	8,5
8.5a.	If respondent answers yes, ask: Who are these groups?						ئەگەر وەڵامدەر بە (بەڵێ) وەڵامی دایەوە، لێی بپرسە: ئەو گروپانە کێن؟	5,a8
8.6.	How much of the clinic's population lives within 1 hour travel time of the clinic? (34)	0 Almost none	1 Less than Half	2 Half	3 More than Half	4 Almost All	چەندێکی ئەو نەخۆشانەی سەردانی بنکە تەندروستیەکە دەکەن سەعاتێك لێوەی دورن؟ (34)	8,6

9. Positive Organizational Structure

9- لایەنە ئیجابییەکانی پێکهاتەی ڕێکخراو

	Question<br>پرسیار	Not at all<br>بە هیچ شێوەیەك	A little bit<br>کەمێك	A moderate amount<br>تا ڕادەیەك- مام ناوەند	A lot<br>زۆر	Do not know<br>نازانم	Question<br>پرسیار	
9.1.	How overworked do you feel in your job? (42)	0	1	2	3	9	تا چەندێك هەست دەکەیت ئەرکی سەرشانت قورسە؟ (42)	9،1
9.2.	To what extent is your boss a good leader? (2)	0	1	2	3	9	تا چ رادەیەك لێپرسراوەکەت سەرکردەیەکی باشە؟ (2)	9،2
9.3.	When you have a problem at work, to what extent do you know who to go to for the answer? (43)	0	1	2	3	9	کاتێك لەکارەکەتدا گرفتێکت دەبێت، تا چ رادەیەك دەزانیت کێ بۆت چارەسەر دەکات؟ (43)	9،3
9.4.	When you have a problem, to what extent is your (boss/supervisor)responsive and has an answer for you? (44)	0	1	2	3	9	کاتێك کە گرفتێکت هەبێت، تا چ رادەیەك ئەو کەسەی دەچیت بۆ لای لە بنکە تەندروستیەکە، بەدەمتەوە دێت؟(44)	9،4
9.5.	How well informed do you feel on things you should know about within the clinic where you work? (45)	0	1	2	3	9	لەناو ئەو بنکە تەندروستیەی کاری تێدا دەکەیت، تا چەندێك هەست دەکەیت زۆر بەباشی ئاگاداری ئەو شتانەیت کە پێویستە بیانزانیت؟ (45)	9،5
9.6.	How positive is your working environment? (13)	0	1	2	3	9	تا چەندێك ژینگەی کارکردنەکەت ئیجابیە؟ (13)	9،6
9.7.	To what extent are there enough learning opportunities offered to you through your clinic? (14)	0	1	2	3	9	تا چ رادەیەك بنکە تەندروستیەکە دەرفەتی باشت بۆ دەرەخسێنێت بۆ فێرکردنی شتی نوێ؟ (14)	9,7

	Question<br>پرسیار	Not at all<br>به هیچ شێوەیەك	A little bit<br>کەمێك	A moderate amount<br>تا ڕادەیەك- مام ناوەند	A lot<br>زۆر	Do not know<br>نازانم	Question<br>پرسیار	
9.8.	To what extent is there a high level of cohesion within this clinic? (17)	0	1	2	3	9	تا چ رادەیەك ئاستێكی بەرزی هەماهەنگی لەناو ئەم بنكە تەندروستیەدا هەیە؟ (17)	9,8
9.9.	To what extent is the communication within this clinic positive and useful (i.e. It is working well.)? (18)	0	1	2	3	9	تا چ رادەیەك لەناو ئەم بنكە تەندروستیەكەدا، پەیوەندییەكی ئیجابی و بەسود هەیە(واتە باشی هەیە) (18)	9,9
9.10.	To what extent does the clinic where you work promote your professional growth? (20)	0	1	2	3	9	تا چ رادەیەك ئەو بنكە تەندروستیەی كاری تێدا دەكەیت پەرە بە گەشەكردنی پیشەییت دەدات؟ (20)	9,10
9.11.	How adaptable is the clinic to solving problems you face in your work? (30)	0	1	2	3	9	تا چەندێك ئەو بنكە تەندروستیەی كاری تێدا دەكەیت گونجاوە بۆ چارەسەری ئەو كێشانەی روبەرووت دەبنەوە لە كارەكەتدا؟ (30)	9,11
9.12	How frustrated are you with the current situation within the clinic where you work? (12)	0	1	2	3	9	تا چەندێك نائومێدیت لە باودۆخی ئێستای بنكە تەندروستییەكە؟ (12)	9,12
9.13	To what extent do staff at this clinic have a high amount of morale? (37)	0	1	2	3	9	تا چ رادەیەك كارمەندانی ئەم بنكە تەندروستیە ورەیان بەرزە؟ (37)	9,13
9.14	How much do you care about the direction and success of this clinic? (41)	0	1	2	3	9	تا چەندێك سەركەوتنی ئەم بنكە تەندروستیەت بەلاوە گرنگە؟ (41)	9,14

10. Sustainability

10-بەردەوامی

	Question<br>پرسیار	No<br>نەخێر	Yes<br>بەڵێ	Maybe<br>لەوانەیە	No Idea<br>نازانم	Question<br>پرسیار	
10.1.	Do you think the step-by-step psychotherapy program will continue after international aid and expert support ends? (48)	1	2	3	4	پێت وایە دوای تەواوبونی پاڵپشت و شارەزایی دەرەکی پرۆگرامی چارەسەری دەرونی هەنگاو بەهەنگاو بەردەوام دەبێت؟	10،1
10.1 a.	If respondent answers NO or MAYBE ask: What do you see as the barriers to sustainability (48a)					ئەگەر وەڵامەکە نەخێر یان (ڕەنگە) بوو،بپرسە: بەلای تۆوە ڕێگرەکان چین؟(48a)	10،1 a

11. Willingness to change

11 - خواستی گۆران

	Question<br>پرسیار	Not at all<br>بە هیچ شێوەیەك	A little bit<br>کەمێك	A moderate amount<br>تا ڕادەیەك- مام ناوەند	A lot<br>زۆر	Do not know<br>نازانم	Question<br>پرسیار	
11.1.	How much would you like to try something new to address the problems in this community? (9)	0	1	2	3	9	تا چەندێك پێت خۆشە شتێکی نوێ بۆ چارەسەری گرفتەکانی ناو کۆمەڵگاکە تاقیبکەیتەوە؟ (9)	11,1
11.2.	How willing are you to try something new if the clinic will get some benefit from it? (10)	0	1	2	3	9	تا چەندێك پێت خۆشە هەوڵی شتێکی نوێ بدەیت بۆ سود گەیاندن بە بنکە تەندروستیەکەت؟(10)	11,2
11.3.	To what extent are you a person who is generally open to change? (11)	0	1	2	3	9	بەگشتی تا چ رادەیەك تۆ کراوەیت بۆ گۆڕان؟ (21)	11,3

12. Other | **12 - شتی تر**

	Question<br>پرسیار	Specify<br>دیاری بکە	Question<br>پرسیار	
12.1.	What are the criteria for selecting mental health staff at this clinic? (21)		مەرجەکانی هەڵبژاردنی کارمەندی تەندروستی دەرونی لەم بنکە تەندروستیەدا چین؟ (21)	12,1

Instructions to interviewer: | **ڕێنمایی بۆ دیمانەساز:**

Thank the person for their time

سوپاسی کەسەکەبکە بۆ ئەو کاتەی بۆ تۆیی داناوە
