# Supplementary material for: Stakeholder perspectives on integration of mental health services into primary care: a mixed methods study in Northern Iraq
Source: Int J Ment Health Syst. 2019 Dec 28;13:75. doi: 10.1186/s13033-019-0330-7 (PMC6935235; doi:10.1186/s13033-019-0330-7)
Supplement: Supplementary file 2 — Additional file 2. Sample qualitative interview guide. [file 13033_2019_330_MOESM2_ESM.pdf]

Qualitative Interview Guide – Providers

ڕێبەری دیمانەی جۆری ـ چارەسازی دەروونی

Instructions to interviewer:

ڕێنمایی بۆ دیمانەساز

1. Greet person.

.1 سلاو کردن.

2. Introduce yourself and explain the study using the consent form.

.2 خۆت بناسێنە و توێژینەوەکە ڕوون بکەوە بە بەکارهێنانی فۆرمی ڕەزامەندی.

3. If person consents and agrees to interview, find a private location (if not already in one).

3. ئەگەر کەسەکە ڕەزامەندی دەربڕی‌و دیمانەکەی قبوڵ کرد، شوێنێکی گونجاو بدۆزەوە بۆ ئەوەی دیمانەکەی تیا سازبکەی.

4. Fill in information at top of notes about:
   - Date
   - InterviewerID number
   - Respondent ID number,
   - Whether this is interview 1 (baseline), or 2 (follow-up).

4. ئەم زانیاریانەی خوارەوە لەسەرەوەی فۆرمی وەڵامەکان تۆمار بکە:
   - بەروار
   - کۆدی دیمانەساز
   - کۆدی وەڵامدەرئایا ئەمە کامە دیمانەیە: یەکەم (لە سەرەتای توێژینەوە) یان دووەم (لەکۆتایی)
   -

5. Ask the respondent the qualitative interview questions. One interviewer asks the questions while the other interviewer records the responses on the qualitative interview notes. Probe until the interviewee says they cannot think of any more to say. Then probe using the specified probes in the qualitative interview to get more specific information.

5. پرسیاری جۆری لە کەسەکە بکە. دیمانەکە لەلایەن دوو کەس ئەنجام دەدرێت: یەکێکیان پرسیار دەکات و دوەم کەسیش وەڵامەکان تۆماردەکات. هەتا دەکرێت چارەسازەکە بە قسە بهێنە تازۆرترین زانیاریت دەست بکەوێت دەربارەی هەر پرسیارێك. ئەمانەی خوارەوە پرسیارەکانن:

Question

پرسیار

5.1. **Tell me about the current situation regarding mental health and mental health care in your community. What are the good things, the challenges or problems, what needs to change about mental health options?**
Be sure to probe on the following:

.5,1 **دەتوانیت پێمان بلێیت تەندروستی دەرونی و خزمەتگوزاری تەندروستی دەرونی لەم ناوچەیەی تۆی لێیت لە ئێستادا چۆنە؟ لایەنە باش و ڕێگرییەکانی چین و پێویستە چی بگۆردرێت دەربارەی ئەو خزمەتگوزارییە تەندرستییە**

- Good things?
- Challenges or problems?
- What needs to change about mental health options?

دەرونیە؟

بە قسەی بهێنە دەربارەی:

- شتە باشەکان
- ڕێگرییەکان و گرفتەکان
- پێویستە چی بگۆردرێت دەربارەی ئەو خزمەتگوزارییە تەندرستییە دەرونیە؟

5.2. **Tell us about any perceptions you believe exist among providers at your clinic about the mental health services offered at your clinic?** (Probe on non-drug mental health services)

.5,2 کارمەندەکانی تر ڕایان چۆنە دەربارەی ئەو خزمەتگوزاریە تەندروستیە دەروونیەی لەم بنکە تەندروستیە پێشکەش دەکرێت؟ (بەقسەی بهێنە دەربارەی خزمەتگوزاری تەندروستی دەرونی بێ دەرمان)

5.3. **Tell us about the working situationat your clinic.When I say *working situation* I mean things such as relationships, supervision, physical space, etc. What are the good things, the challenges or problems, what needs to change about the working situation?**
Be sure to probe on the following:

- Good things?
- Challenges or problems?
- What needs to change about the working situation?

.5,3 دەتوانیت باسی بارودۆخی کارکردن لەم بنکەیەمان بۆ بکەیت؟ مەبەستمان لە بارودۆخی کارکردن ئەوەیە بۆ نمونە،پەیوەندی کارمەندەکان لەگەڵ یەکتری، سەرپەرشتیکردن، بونی جێگا، هتد. لایەنە باش و ڕێگرییەکانی چین و پێویستە چی بگۆردرێت دەربارەی ئەو خزمەتگوزارییە تەندرستییە دەرونیە؟

بە قسەی بهێنە دەربارەی:

- شتە باشەکان
- ڕێگرییەکان و گرفتەکان
- پێویستە چی بگۆردرێت دەربارەی ئەو خزمەتگوزارییە تەندرستییە دەرونیە؟

5.4. **I'd like to have you tell me about your experiences with step-by-step psychotherapies. What are the positive things, the gaps or challenges, what needs to be changed about step-by-step psychotherapy?**
Be sure to probe on the following:

- Positive things?
- Gaps or challenges that still need to be resolved?
- What needs to change about step-by-step psychotherapy?

.5,4 پێمان خۆشە باسی ئەزموونی خۆتمان بۆ بکەیت دەربارەی چارەسەری دەروونی هەنگاو-بە-هەنگاو. چ شتێکی بەلاتەوە باش بوو، چ شتێکی بەلاتەوە باش نەبوو، هەروەها پێت باشە چ گۆرانکاریەك لەو چارەسەرەدا بکرێت؟

بە قسەی بهێنە دەربارەی:

- شتە باشەکان
- ڕێگرییەکان و گرفتەکان
- پێویستە چی بگۆردرێت دەربارەی ئەو

خزمەتگوزارییە تەندرستییە دەرونیە؟

5.5. **Now I would like to ask you some different questionsabout step-by-step psychotherapy.**

ئێستا دەمەوێت پرسیار دەربارەی چەند لایەنێکی ئەم چارەسەری هەنگاو بە هەنگاوەت لێ بکەم. .5,5

5.5.1 **Tell me specifically about the feasibility of step-by-step psychotherapy. When I say *feasibility* I mean whether step-by-step psychotherapy can be carried out with the available resources. These resources include appropriately trained personnel, money, time, buildings and furniture, technology, communication.**
(Probe on:
- What makes it more feasible?
- What makes it less feasible?
- What needs to change to make it more feasible?)

دەتوانیت بەڕێزتان باسی ئەوەمان بۆ بکەیت ئایا ئەم چارەسەری دەرونی هەنگاو بەهەنگاوەتا چ ڕادەیەك عەمەلیە؟ مەبەستم ئەوەیە کە ئایا دەکرێت ئەم بەرنامەیە بە ئیمکاناتی ئێستا پیادە بکرێت؟ ئەم ئیمکاناتانە بریتین لە: کارمەندی مەشقپێکراو بە شێوەیەکی گونجاو، پارە، کات، بینایە، کەلوپەل وەك مێز و کورسی هتد..، و هۆیەکانی پەیوەندیکردن. .5,5,1

بەقسەی بهێنە دەرباری:
- چی وا ئەکات زیاتر عەمەلی بێت؟
- چی وا ئەکات کەمتر عەمەلی بێت؟
- پێویستە چی بگۆردرێت بۆ ئەوەی زیاتر عەمەلی بێت؟

5.5.2. **Tell me specifically about the acceptability of step-by-step psychotherapy. When I say *acceptability* I am talking about the perception that step-by-step psychotherapy is agreeable, palatable, and/or satisfactory.**
(Probe on:
- what makes it more acceptable?
- What makes it less acceptable?
- What needs to change to make it more acceptable?)

ب. دەتوانیت بەڕێزتان باسی ئەوەمان بۆ بکەیت ئایا ئەم بەرنامەیە تا چ ڕادەیەك مەقبوولە؟ مەبەستم ئەوەیە کە ئایا ئەم چارەسەری دەرونی هەنگاو بەهەنگاوە تا چ ڕادەیەك قبوول دەکرێت. (.5,5,2

بەقسەی بهێنە دەرباری:
- چی وا ئەکات زیاتر مەقبول بێت؟
- چی وا ئەکات کەمتر مەقبول بێت؟
- پێویستە چی بگۆردرێت بۆ ئەوەی زیاتر مەقبول بێت؟

5.5.3. **Tell me specifically about the sustainability of step-by-step psychotherapy. When I say *sustainability* I am talking about the extent to which you think step-by-step psychotherapy will continue after internationalfunding andsupport**

باسی بەردەوامبوونی چارەسەری دەرونی هەنگاو بە هەنگاوم بۆ بکە. مەبەستم ئەوەیە ئەگەری بەردەوامبوونی ئەم چارەسەری هەنگاو بە هەنگاوە .5,5,3

	**from international experts is terminated.**<br>(Probe on:<br>• What makes it more sustainable? What makes it less sustainable?<br>• What needs to change to make it more sustainable?)	چۆنە ئەگەر هاوکاری نێونەتەوەیی نەمێنێت؟<br>بەقسەی بهێنە دەرباری:<br>• چی وا ئەکات زیاتر بەردەوام بێت؟<br>• چی وا ئەکات کەمتر بەردەوام بێت؟<br>• پێویستە چی بگۆردرێت بۆ ئەوەی زیاتر بەردەوام بێت؟	
5.5.4 .	**Tell me specifically about the appropriateness of step-by-step psychotherapy. When I say *appropriateness* I am talking about your perceptions on whether step-by-step psychotherapy is a good way to address the mental health problems of your clients.**<br>(Probe on:<br>• What makes it more appropriate?<br>• What makes it less appropriate?<br>• What needs to change to make it more appropriate?)	د. دەتوانیت بەڕێزتان باسی گونجاوی ئەم چارەسەری هەنگاو بە هەنگاوە بکەیت؟ مەبەستم ئەوەیە بەڕای ئێوە ئایا ئەم چارەسەرە باشە بۆ چارەسەرکردنی گرفتی نەخۆشەکانت؟<br>بەقسەی بهێنە دەرباری:<br>• چی وا ئەکات زیاتر گونجاو بێت؟<br>• چی وا ئەکات کەمتر گونجاو بێت؟<br>• پێویستە چی بگۆردرێت بۆ ئەوەی زیاتر گونجاو بێت؟	.5,5,4
5.6.	**Tell us about any perceptions you believe exist among your colleagues about step-by-step psychotherapy?**	هاوکارەکانت ڕایان چیە دەربارەی چارەسەری دەرونی هەنگاو بە هەنگاو؟	.5,6
5.7.	**Tell us how you came to be recruited to provide step-by-step psychotherapy** (Probe on:<br>• How were you chosen?<br>• What were you told?<br>• By whom?<br>• Your thoughts on the recruitment process?)	چۆن بەڕێزتان دەستنیشان کران بۆ بەشداری کردن لە خولی راهێنانی چارەسەری دەرونی هەنگاو بە هەنگاو؟ (بەقسەی بهێنە دەربارەی: چۆن هەڵبژێردرایت؟ چیان پێ گوتیت؟ لەلایەن کێوە؟ ڕات چیە دەربارەی پرۆسەی دەستنیشان کردنەکە؟)	.5,7
5.8.	**What are the challenges to implementing step-by-step psychotherapy? What are the things that make it easier to implement step-by-step psychotherapy?**	ئەو بەربەستانە چین کە ڕێگرن لەبەردەم جێبەجێ کردنی چارەسەری دەرونی هەنگاو بە هەنگاو ؟ ئەو هۆکارانە چین کە جێبەجێ کردنی ئاسانتر دەکەن؟	.5,8
5.9	**In this area, we're seeing difficulties in maintaining the program and how it will continue after outside support ends. What**	هەست دەکەین لەم ناوچەیەدا قورسیەك هەیە لە	5,9

are all the problems that in your opinion have affected the implementation of the program?

مانەوەی بەرنامەکە و چۆنێتی بەردەوام بوونی دوای تەواوبونی پاڵپشتی دەرەکی. بە بۆچونی تۆ چین ئەو کێشانەی کە کاریگەریان هەبووە لەسەر جێبەجێکردنی بەرنامەکە؟

5.10 **What are all the changes that need to be made to ensure that the program will continue here after outside supports end?**

5,10 **ئەو گۆرانکایانە چین کە پێویستە بکرێن بۆ ئەوەی دڵنیابین کە بەرنامەکە لەم ناوچەیەدا بەردەوام دەبێت دوای تەواوبوونی پاڵپشتی دەرەکی؟**

5.11 **Is there anything else you think is important to tell us about mental health services?**
(Probe on:
- Barriers
- Facilitators
- Strong beliefs
- Cultural considerations
- Logistical issues)

.5,11 **بەڕای ئێوە هیچ خاڵێکی تر هەیە کە گرنگ بێت دەربارەی خزمەتگوزاری تەندروستی دەروونی؟ (بەقسەی بهێنە دەربارەی: بەربەستەکان، ئاسانکارییەکان، هۆکاری کەلتووری، بیروباوەڕی توند، هۆکاری لۆجیستی)**

5.12. **Is there anything else you think is important about step-by-step psychotherapy in particular?**

.5,12 **هیچ خاڵێکی تایبەتهەیە کە گرنگ بێت دەربارەی چارەسەری دەرونی هەنگاو بە هەنگاو و بتەوێت پێمان بڵێیت؟**

Instructions to interviewer:

<u>ڕێنمایی بۆ دیمانەساز</u>

6. Both interviewers read through the notes with the interviewee still present. If anything is not clear ask for clarification and correct your notes as necessary.

.6 هەردوو دیمانەسازەکە وەڵامەکانبخوێننەوەلەوکاتەیکە کەسەکە هێشتالاتاندانیشتووە.

ئەگەرهەرخاڵێکڕوونوئاشکرانەبوو،دووبارەپرسیاریلێبکەنەوەووەڵامەکانبەگوێرەیپێویستڕاستبکەنەوە.

7. Ask if interviewee has anything to add. Add to the interview notes as needed.

.7 بپرسە: **وەکدواقسە،هیچیترتماوەبیڵێیت؟**

(ئەگەرهەبووئەواتۆماریبکەن).

8. Ask if you can return if necessary (record when and where you can return in your notes).

.8 بپرسە: **ئایادەتوانیندووبارەدیمانەتلەگەڵبکەینئەگەر پێویست بوو؟**

(ئەگەررازیبوو،لەسەرپشتیفۆرمیوەڵامەکانکاتوشوێنەکەبنووسەک

ەگونجاوبێتبۆهەردوولاتان).

9. Thank person and leave.

9. سوپاسیبکەوکۆتاییبەدیمانەکەبهێنە.
